# Activity Response to Climate Seasonality in Species with Fossorial Habits: A Niche Modeling Approach Using the Lowland Burrowing Treefrog (*Smilisca fodiens*)

**DOI:** 10.1371/journal.pone.0078290

**Published:** 2013-11-11

**Authors:** Alondra Encarnación-Luévano, Octavio R. Rojas-Soto, J. Jesús Sigala-Rodríguez

**Affiliations:** 1 Departamento de Biología, Universidad Autónoma de Aguascalientes, Aguascalientes, Aguascalientes, México; 2 Red de Biología Evolutiva, Instituto de Ecología, A. C., Xalapa-Enríquez, Veracruz, México; University of Sao Paulo, Brazil

## Abstract

The importance of climatic conditions in shaping the geographic distribution of amphibian species is mainly associated to their high sensitivity to environmental conditions. How they cope with climate gradients through behavioral adaptations throughout their distribution is an important issue due to the ecological and evolutionary implications for population viability. Given their low dispersal abilities, the response to seasonal climate changes may not be migration, but behavioral and physiological adaptations. Here we tested whether shifts in climatic seasonality can predict the temporal variation of surface activity of the fossorial Lowland Burrowing Treefrog (*Smilisca fodiens*) across its geographical distribution. We employed Ecological Niche Modeling (ENM) to perform a monthly analysis of spatial variation of suitable climatic conditions (defined by the July conditions, the month of greatest activity), and then evaluated the geographical correspondence of monthly projections with the occurrence data per month. We found that the species activity, based on the species' occurrence data, corresponds with the latitudinal variation of suitable climatic conditions. Due to the behavioral response of this fossorial frog to seasonal climate variation, we suggest that precipitation and temperature have played a major role in the definition of geographical and temporal distribution patterns, as well as in shaping behavioral adaptations to local climatic conditions. This highlights the influence of macroclimate on shaping activity patterns and the important role of fossorials habits to meet the environmental requirements necessary for survival.

## Introduction

Ecological niches can be defined as the set of conditions within which a species can maintain populations without immigrational input [1], and constitute an important constraint in the distribution of species [2], [3]. Evolutionary studies of ecological niches through modeling methods are now frequently used due the critical dimension of the ecological requirements in the evolutionary biology of organisms [4]. A finding derived from this field of study is that ecological niches represent long-term stable constraints on geographical distributions of species [4]–[6]. Geographical patterns of environmental conditions within the distribution of species [7] influence speciation or extinctions rates of organisms modifying their life histories as a consequence [8]. For example, some species of mammals faced past climate change events by tracking niches spatially, changing their distributional patterns while retaining their niches [5]. Furthermore, it has been shown that this “niche tracking” applies also across seasonal shifts in distributions [9]. Some neartic-neotropical migrant species of birds move along their distributional range following a set of climatic conditions year-round [10]. This suggests that behavior may evolve due to changing climates [11] to maintain the organism's niche along space and time, and thus avoiding extinction.

But what about organisms with a low dispersal ability like amphibians? Could it be signs of behavioral adaptations due to the effects of seasonal climate within the distributional range of these ectothermic organisms? Despite the high sensitivity of amphibians to environmental variables [12]–[16], movements to track niches would not be expected as in mammals and birds due their low dispersal ability [12], [13], [17]. Therefore, we have to turn our attention to the physiological [14], [15] and behavioral strategies [14] that amphibians have evolved in order to succeed in challenging climates. For example, it has been shown that the ecological success of anurans in different thermal environments is largely due to physiological adaptations [14], [15], [18]. However, it is also known that the range of adjustment (i.e. acclimation) for tropical and temperate species is about the same [18]. Therefore, there must be other traits, like behavior, that play a role in this success in different thermal environments.

Among amphibians, the Hylid frog family is widely distributed around the world [19]. Within the New World most of the species of the clade inhabit tropical regions due to the phylogenetic conservatism of tolerance to extreme seasonality of most species in the group [20] but five of these species (out of 668) reach temperate zones in the Neartic region with marked climatic seasonality [20], [21]. Moreover, the Lowland Burrowing Treefrog (*Smilisca fodiens*) has the northernmost distribution of the family; its current distribution encompasses a significant climatic gradient, from the desert scrub in south-central Arizona [22], southward along the Pacific coast through western Sonora, into the thorn forest, tropical deciduous and semi-deciduous forest of Sinaloa, Nayarit and Colima, in Mexico [23]. It also inhabits inland patches of these same vegetation types from central Jalisco to northern Michoacán. This species evolved a fossorial habit that gives it several ecological advantages [24]. It is known, especially by local studies at northern populations, that *Smilisca fodiens* spends a period of the year inside underground burrows, until the climatic conditions trigger a brief and explosive period of surface activity [22]. Our goal is to address the reasons that allowed *Smilisca fodiens* to reach such a high latitudinal range, far above the northern limit of the remaining hylids. We suggest that evolving a fossorial behavior allows this species to inhabit temperate regions while retaining its climatic niche.

In this study, using coarse-scale ecological context of species niches [1], we test whether shifts in climatic seasonality can predict the temporal variation of surface activity of *Smilisca fodiens* across its geographical distribution. We model the ecological niche of the species based on the month with the most suitable conditions for the species activity (i.e. July) and project it on the climatic conditions of the remaining months of the activity period (defined by the occurrence data). We evaluate the geographical correspondence of monthly projections with the occurrence data per month and discuss how a behavioral trait associated to fossorial activity can favor the conservation of the climatic requirements of a species with low dispersal ability inhabiting a marked climatic gradient.

## Materials and Methods

### Distributional and environmental data

We compiled locality occurrence records from three sources: biological collections (Global Biodiversity Information, GBIF; Herpetological Collection Networks, HerpNET; Unidad Informática para la Biodiversidad, UNIBIO, UNAM); published literature [22]; and experts field surveys (see Acknowledgments). Because *S. fodiens* is fossorial, we assumed that the records ensured that the collected organisms were found on the surface in suitable environmental conditions. We used records with geographic information (latitude-longitude); and those with no geospatial information were georeferenced using BioGeomancer (http://www.biogeomancer.org) and the Georeferencing Calculator (http://manisnet.org/gci2.html). Each locality record was verified in ArcView 3.2 [25].

We employed a set of five variables for each of the analyzed months (four climatic and one topographic). The layers of maximum and minimum monthly temperature (Tmin and Tmax), monthly mean temperature (Tmean) and monthly total precipitation (Prec) were obtained from the WorldClim project (http://www.worldclim.org/) and are the result of the interpolation of monthly averages from weather stations throughout the world, from 1950 to 2000 [26]. Slope was obtained from the digital elevation model GTOPO20, available at the EROS data center (http://eros.usgs.gov/). Resolution of all layers was 30 arc-seconds (∼1 km^2^). The selection of the climatic variables was based on their relevance for amphibians [8], [15]–[17]. We included slope in the dataset to improve the performance of the model and because it is not a variable directly correlated with precipitation and temperature [27].

We modeled the climatic niche of the month of July, when the species is most active (i.e. month with most occurrence data points), assuming that this month meets the climatic conditions that are the most suitable for species activity. Of the 95 occurrence data points used for the model, only four were outside the temporal interval (1950–2000) of the climatic layers. Thus, we expect no effects due to the climate variation outside these five-decades that compromise the reliability of the potential niche obtained for July. Finally, the climatic niche was then projected on the climatic conditions of the remaining months of the period of activity: May, June, August, September, October, November and December (months in which we found at least one occurrence data point).

### Ecological niche modeling

We employed two automatic learning algorithms: the Genetic Algorithm for Rule-set Prediction (GARP) and the maximum entropy approach (Maxent). For GARP models we used a desktop version which operates under a stochastic process where classifiers (e.g., truncation, point changes, crossing-over, among other rules) compete to select solutions that identify the presences and the pseudoabsences [28]. The algorithm overlay *n* simulations, where the result is an index of how favorable the climatic conditions are to species requirements [9], [29]. The algorithm determines all possible localities in a grid with similar environmental characteristics, generating a map of the potential distribution area of the species in a geographical space [9], [30]. This is the projection of the fundamental niche into the geographical space [3]. To optimize model performance in GARP, 100 replicates were carried out, based on random subsets of the 80% of the occurrence data points. To force GARP models to be general and to minimize overfitting, we used the best subsets procedure [29].

Maxent fits a probability distribution for occurrence of the species to the set of pixels across the study region subject to the appropriate constraints. In ecological niche modeling these constraints are the expected value of each feature, which should match its empirical average. We assigned 80% of the occurrence data points to formulate the model parameters. For Maxent models we used the default parameters (i.e., no random subsampling, regularization multiplier  = 1, maximum iterations  = 500, 10,000 background points, convergence limit  = 10^−5^).

Performance evaluation for both algorithms was done based on the AUC (Area Under the Curve) ratios [31] with the tool for Partial ROC [32]. For this test we employed random subsets of 20% of the occurrence data points which were selected prior to developing the model. With a threshold of acceptable omission error of 5% the AUC ratio were highest for GARP and lower for Maxent (1.55 and 1.45, respectively). Moreover, GARP AUC ratio was significantly higher than null expectations (p<0.001) but Maxent did not achieve statistical significance based on the simpler counts of numbers of replicates with AUC ratios of >1 (p = 0.074). Thus, due to the poor performance of the Maxent model, the subsequent analyses were based on GARP results. We imported GARP predictions into ArcView 3.2 and then summed the resulting 10 grids to create a surface summarizing model agreement, with values ranging from 0 to 10 as integers. For visualization of these results, we present maps showing various thresholds of concordance among models: (1) the distribution of pixels with suitable conditions predicted by at least 2/10 models; (2) pixels predicted by 3 to 5/10 models; (3) pixels predicted by 6 to 8/10 models; and (4) pixels predicted by 9 to 10/10 models.

**Figure 1:**
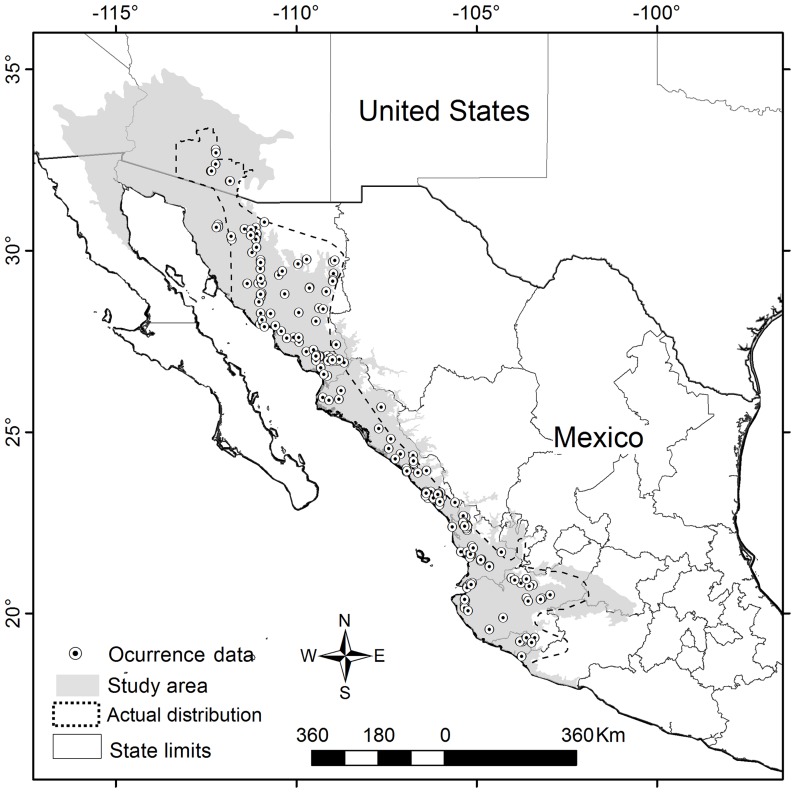
Occurrence data and area predicted by temporal analysis. Unique occurrence data points recorded for the species (*dots in the map*) that delimit the actual distribution (*dashed line*, expert map). Only the July occurrence data was used to model performance. Because ENM generally over predicts since it does not consider biological, historical and geographical factors, the predicted area generated by models was edited based on the geographical limits of the terrestrial ecoregions proposed for Mexico and for United States (*gray shaded area*).

The distributions obtained by ENM generally over predict because the model does not consider the factors that may have limited biologically, historically and geographically the occupation of such niches [33], [34]. In order to avoid this overprediction, to highlight biogeographical patterns and because ecoregions might determine the distributional limits of species [35], the models were edited based on the geographical limits of terrestrial ecoregions (Fig. 1) [36], [37], and we used only prediction areas contained in ecoregions in which there was at least one record of the species [34].

### Analysis of climatic niche

The temporal variation of suitable climatic conditions throughout the area of distribution was evaluated based on both the degree of correspondence between the monthly projections and occurrence data, and the estimation of the percentage of predicted area for each month. We determined the amount of monthly occurrence data that coincided with the monthly projections and estimated the kappa coefficient [38]. To obtain this coefficient, and in order to catalog July occurrence data as presences, we assumed that the degree of agreement between the July prediction and the rest of the monthly projections would be stronger in months adjacent to July and weaker as it departs from this month. We built a confusion matrix to determine the agreement between the July prediction and the monthly projections. To estimate the Kappa coefficient we considered the presence of suitable conditions as the area in which at least 6 out of the 10 best models indicate the potential presence of the optimal climatic conditions in GARP. This threshold allows estimating the Kappa coefficient in most of the months of activity. Coheńs Kappa values were estimated with the *kappa2* function in the *irr* package [39] using the R statistical software [40]. For each monthly Kappa statistic value, the relative strength of agreement was assigned [38] and the significance of the statistical test associated with Kappa was set at a *p*-value less than 0.05. The amount of predicted area was estimated based on the thresholds of agreement described above and presented as percentages of number of pixels.

To describe the environmental space for species activity, we analyzed the ranges of precipitation and minimum temperature (variables that explain most of the occurrence data variation based on a Principal Component Analysis –not shown–) that are suitable for the species activity based on the climatic information of the occurrence data. Because the occurrence data points of July are widely and evenly spread along the distribution area of the species, we used them to demonstrate that the climatic conditions suitable for activity spatially vary throughout the year. Thus we analyzed the climate ranges of July points both for the dry season, when activity is not reported (January to April), and for the season in which the period of activity is favored (May to December), and then were compared with the climate ranges of remaining months of the activity period. For this we used the *Spatial Analyst* extension of ArcView 3.2. Finally, we compared the monthly variation in the precipitation and minimum temperature ranges performing a Mann–Whitney U-test (considering only those months with more than 10 occurrence data points: June, August and September) with the *wilcox.test* function in the *stats* package [40].

## Results

**Figure 2:**
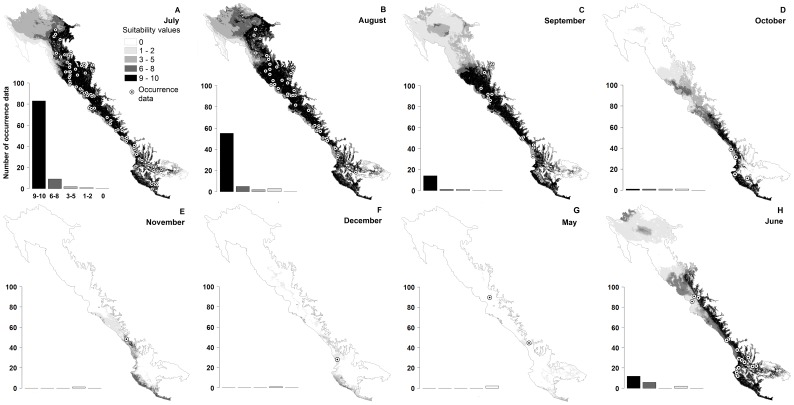
Geographical distribution of suitable climatic conditions temporally reconstructed using GARP algorithms. The climatic niche of July was projected to the climatic conditions of May, June, August, September, October, November, and December. We present the geographical projection for July (*A*) and for the remaining months of the period of activity (*B* to *H*). The *black shading* from pale to dark indicates the concordance of the ten best models to predict suitable conditions. The *dots* indicate the occurrence data per month. The amount of those that is coincident with the monthly projections is shown in a bar graph at the lower left side of each map.

**Figure 3:**
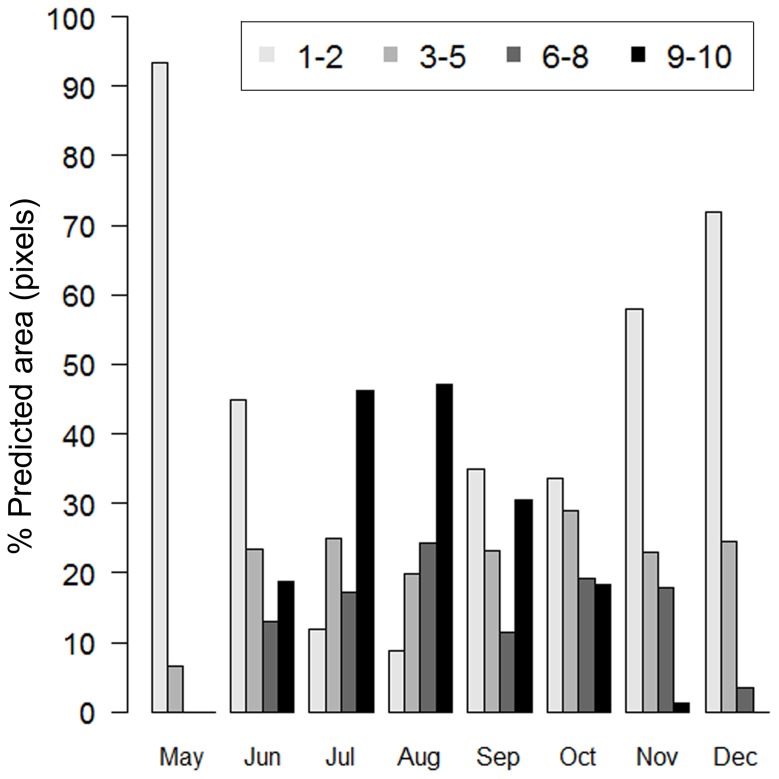
Graphical representation of the temporal variation of the area predicted per month. Each bar corresponds to a threshold in the distribution of pixels predicted as presence of suitable conditions according to model concordance and cumulative probability (*indicated as percentage*). Note the decrease in area as it departs from July until June, when we observed a marked recovery of suitable area.

We found that the geographic distribution of suitable climatic conditions for the activity of the species varies temporally (Fig. 2). We observed a decrease in the amount of area with suitable climatic conditions as it departs from July (Fig. 3). The suitable climatic conditions disappeared first in the northern, then the central and lastly in the southern portions of the range, until January when there is no suitable climatic conditions available (Fig. 2). In June, coinciding with the onset of the rainy season, we observed a notable recovery in the amount of area with suitable climatic conditions (Fig. 3) starting in the south towards the central and northern portions of the range (Fig. 2).

The occurrence data for each month of the activity period (i.e. May to December) showed a high geographical correspondence with the variation of suitable conditions throughout the study region (i.e. monthly projections, see graphs in Fig. 2). Even for May, November and December, and despite the mismatch between the consensus maps and the occurrence data points, we observed a coincidence between those months and the latitudinal range predicted as suitable for those months. We found that the degree of agreement between the July prediction and the rest of the monthly projections is stronger in months closer to July and weaker as it departs from that month (Table 1).

**Table 1:** Summary of Kappa statistic.

	Kappa value	Strength of agreement	Z	*p*-value
May	0	-	NA	NA
June	0.057	Slight	1.67	0.095
August	0.852	Almost perfect	8.39	0
September	0.195	Slight	3.21	0.001
October	0.048	Slight	1.53	0.125
November	0.001	Slight	0.258	0.796
December	0	-	NA	NA

For each analyzed month, we present the kappa values and the strength of agreement assigned as proposed by Landis and Koch [38]. The kappa coefficient was evaluated based on two predictors (July prediction and monthly projection) and 95 occurrence data from July. The kappa coefficient evaluates the degree of agreement between predictors to catalog the occurrence data; thus, in months in which the area of prediction is little or null, the evaluation cannot be generated and it is indicated as NA.

**Figure 4:**
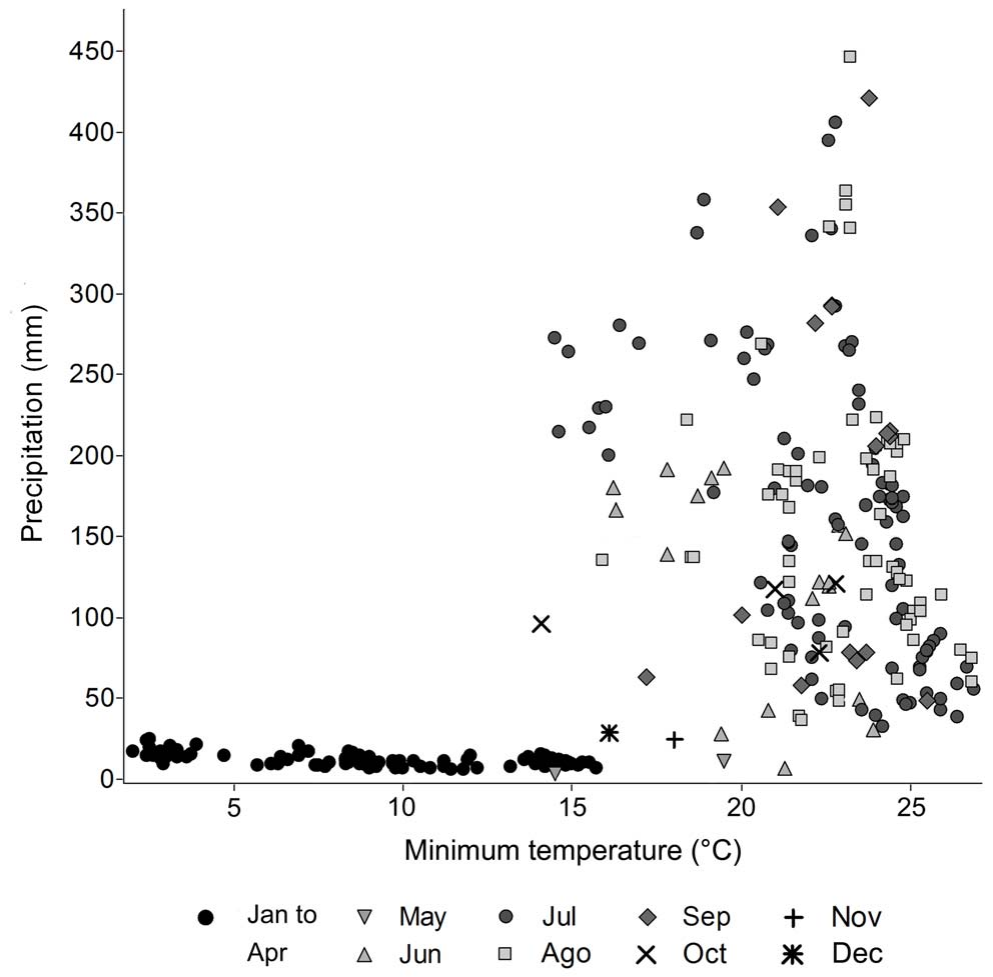
Environmental space of the niche. It was constructed using the information of precipitation and minimum temperature of the monthly occurrence data. Note that the occurrence data of July, when plotted to months without activity (*indicated by black dots*), fall completely outside of the area of the climatic range of suitable conditions in the period of activity (*indicated by all other symbols*).

**Figure 5:**
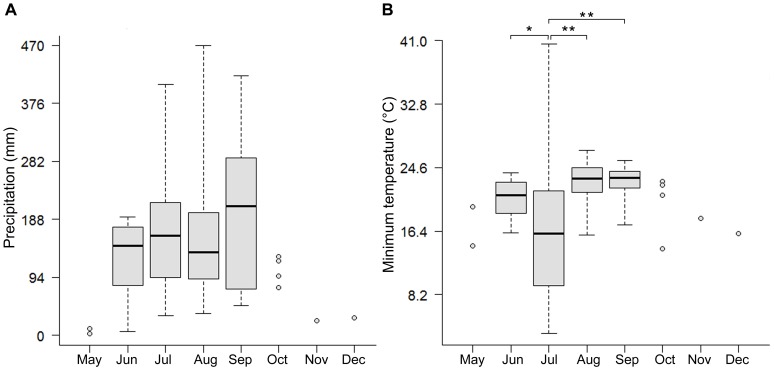
Variation of precipitation and minimum temperature ranges. We present a median comparison performed with a Mann-Whitney *U*-test between the occurrence data of July and the months with more than ten occurrence data. A) For precipitation no significant differences exist between the July median (161.0) and the ones calculated for June, August and September (145.5, 135.0, 209.0 mm, respectively, p>0.05). Contrarily, B) for minimum temperature we found significant differences between the July median (16.1) and those for June, August and September (21.1, 23.2, 23.3°C, respectively, * p<0.05, ** p<0.001).

In the analysis of the environmental space we found that the climatic conditions suitable for activity spatially vary throughout the year. The environmental space of the dry season (January to April) did not match that described for the activity period (May to December) (Fig. 4). Finally, we found no significant differences between the precipitation medians observed between the July median (161.0) and the ones calculated for June, August and September (145.5, 135.0, 209.0 mm, respectively, *p*>0.05; Fig. 5A). Contrary, for minimum temperature we found significant differences between the July median (16.1) and those for June, August and September (21.1, 23.2, 23.3°C, respectively, * *p*<0.05, ** *p*<0.001; Fig. 5B).

## Discussion

We found that the activity of the species outside burrows is predicted by climatic conditions (Fig. 4) and that it is influenced by the latitudinal variation of climate due to seasonal effects, typical of the temperate area in which the species inhabits (Fig. 2). It seems that the distributional range of *Smilisca fodiens* has a range of climatic conditions wider that the species can tolerate, so it can be suggested that the species evolved behavioral [11] instead of physiological traits [11], [15], [41], [42] to maintain its ecological niche, avoiding the extreme climatic conditions that characterize its temperate-dry geographic distribution.

Several local-scale studies in fossorial anurans have shown the influence of microclimatic conditions over the explosive surface activity of individuals [17], [43]. In this large-scale analysis, the surface activity of this fossorial species is predicted on the basis of the temporal and geographical variation of suitable climatic conditions throughout its distributional range. The gradual reduction of the area with suitable conditions towards the southern portions of the distributional range, as it departs monthly from July, reveals the relatively straightforward relationship among the climate variables, latitude [44] and seasonality.

The fossorial behavior is a common strategy among temperate anurans [17], [45] to cope with harsh environmental conditions. The physical similarities among fossorial species of diverse families suggest behavioral convergence [24]. Environmental variables induce phenotypic plastic shifts in organisms which, under some climatic scenarios, are selected for the evolution of behavioral and ecological traits [46]. Consequently, the link we found between the seasonality in the activity of *Smilisca fodiens* and the annual latitudinal variation of suitable conditions could reflect a behavioral adaptation in order to retain its physiological tolerance ranges. For instance, amphibians from xeric environments rely on rainy conditions to avoid desiccation due to the high permeability of the skin [47]. Based on our results we suggest that the behavioral response of the Lowland Burrowing Treefrog has favored the conservation of the climatic requirements in the species under current climatic conditions. This finding highlights the importance of behavioral traits over physiological adaptations not only for this fossorial species but, more generally, for temperate ectotherms with wide distributional ranges, as it has been demonstrated in snakes [43], [48].

Local-scale studies of fossorial anurans [24], [45] have shown the strong influence of climate over fossorial habits, specifically in the entrance, emergence, or time spent within the refuges [17], [24], [47]; however, our results might indicate that macroclimatic variables play a prominent role in such adaptations, as the environmental space of the dry season and of the activity period are remarkably distinct (Fig. 4). It seems that the precipitation (Fig. 5A) is the variable that better explains the environmental space constraint for seasonal activity. Furthermore, looking at the U-test it is clear that precipitation alone cannot be the single factor that makes July optimal for the species activity, rather a combination of this with other environmental variables, as it happens with minimum temperature (Fig. 5B). Several studies in ectotherms have focused on thermal responses for adaptation [14], [15], [18], [41][Navas, 2008 #6], and recently, those studies have become common due to the uncertainty of how species respond biologically to the increase of air temperature projected in next few decades [49]. Our results suggest that it will be critically important to assess the biological impact of changes in precipitation patterns on species whose ecological requirements are similar to those of *Smilisca fodiens*. Such changes appear to be a strong selective force in species adaptation, as it has been shown when the response rate of some traits (i.e. behavioral, physiological) increase its selective values at the optimums of the environmental tolerance range of species, particularly, when there is an increase in the spatial and temporal heterogeneity of environment between generations [42].

Based on the importance of precipitation on the activity of this species, the increase in aridity during the Pleistocene along its geographical range, could have favored its differentiation within the *Smilisca* group [50]. Moreover, the evolution of behavioral responses to these past climate changes events could anticipate how ongoing climate change processes would impact the distribution [51] and periods of activity of species. Considering that increasing global surface temperatures due to ongoing climate change are likely to lead to changes in precipitation patterns, and that dry and arid areas could become more arid [52], we assume that fossorial anurans would be more severely affected than other members of the group. These changing patterns in climate calls for renewed efforts for adaptation [52], although we do not know if the species can respond physiologically to such rapid changes [53], we conclude that *Smilisca fodiens* will face a reduction in its already restricted activity period for feeding and breeding, and that metabolic responses during dormancy in cold periods can be compromised, affecting the survival of the species. In this context, it will be particularly important that future studies address the possible impact of the shifts in climatic patterns over fossorial anurans.

The important role that climatic conditions have on the distribution patterns of anurans is well known, but the role of spatial and temporal climatic variation in the activity of fossorial anuran species is poorly understood. Despite this analysis focusing on a single species, the life history traits and the evolutionary history of the Lowland Burrowing Treefrog allows us to project our findings to other ectothermic organisms with low dispersion ability. We expect that approaches based on the analyses of ecological niches can contribute and enhance the understanding of current patterns and its evolutionary processes. All of these as part of a more general theory of seasonality of the ecological niches.

## Supporting Information

Acknowledgments S1 **Collections and institutions included in HerpNET and GBIF that provided historical occurrence.**
(DOCX)
