# Supplementary material for: Activity Response to Climate Seasonality in Species with Fossorial Habits: A Niche Modeling Approach Using the Lowland Burrowing Treefrog (Smilisca fodiens)
Source: PLoS One. 2013 Nov 11;8(11):e78290. doi: 10.1371/journal.pone.0078290 (PMC3823916; doi:10.1371/journal.pone.0078290)
Supplement: Acknowledgments S1 — Collections and institutions included in HerpNET and GBIF that provided historical occurrence. (DOCX) [file pone.0078290.s001.docx]

**Acknowledgments S1**

We thank to the collections and institutions that provided historical occurrence data via HerpNET and GBIF data portal (http://www.herpnet.org, http://www.gbif.org, respectively) accessed on September 23th 2011. American Museum of Natural History, New York; Herpetology Collection, California Academy of Sciences, California; Arizona State University, Arizona; Brigham Young University, Hawaii; Herpetology Collection, California Academy of Sciences, San Francisco; Amphibians and Reptiles Collection, Cornell University Museum of Vertebrates, Ithaca; Colección Nacional de Anfibios y Reptiles, Instituto de Biología de la UNAM, Distrito Federal; Natural History Museum, University of Kansas, Lawrence; Los Angeles County Museum of Natural History, Los Angeles; Museum of Natural Science, Louisiana State University, Baton Rouge; Michigan State University Museum, East Lansing; Collection Herpetology, Museum of Comparative Zoology Harvard University, Cambridge; Herpetological Collection, Museum of Vertebrate Zoology, University of California, Berkeley; Herpetological Collection, Smithsonian National Museum of Natural History,Washington; Santa Barbara Museum of Natural History, Santa Barbara; Vertebrate Collection, Texas Cooperative Wildlife Collection, College Station; Amphibian and Reptile Collection, University of Arizona, Tucson; Colección de Anfibios y Reptiles, Museo de Zooología, Facultad de Ciencias, UNAM, Distrito Federal; Herpetology Collection, University of Colorado Museum of Natural History, Boulder; Herpetology Collection, The University of Texas at El Paso, El Paso.
